# Influence of Preheating Temperature on the Microstructure and Mechanical Properties of 6061/TA1 Composite Plates Fabricated by AFSD

**DOI:** 10.3390/ma16176018

**Published:** 2023-09-01

**Authors:** Wei Gong, Yidi Li, Ming Zhang, Hui Wang, Qinglin Liu, Ziming Zeng, Kuo Ma, Biaobiao Yang, Ruilin Lai, Yunping Li

**Affiliations:** 1 State Key Laboratory for Powder Metallurgy, Central South University, Changsha 410083, China; gongwei1999@csu.edu.cn (W.G.); liiidi@csu.edu.cn (Y.L.); zhangming717@csu.edu.cn (M.Z.); wanghuiii@csu.edu.cn (H.W.); silin_link@csu.edu.cn (Q.L.); zndxzzm@csu.edu.cn (Z.Z.); makuo@csu.edu.cn (K.M.); 133701033@csu.edu.cn (R.L.); 2 IMDEA Materials Institute, C/Eric Kandel 2, Getafe, 28906 Madrid, Spain; 3 Department of Materials Science, E.T.S. de Ingenieros de Caminos, Polytechnic University of Madrid, 28040 Madrid, Spain

**Keywords:** 6061/TA1 composite, additive friction stir deposition, preheating, bonding performance, finite element analysis

## Abstract

In this study, composite plates of 6061/TA1 were successfully manufactured using additive friction stir deposition (AFSD). The impact of preheating temperatures (room temperature, 100 °C, 200 °C) on the interfacial microstructure and interface mechanical properties at various deposition zones was studied. The results showed that as the preheating temperature increased or when the deposit zone shifted from the boundary to the center, the diffusion width of Al and Ti increased, accompanied by an increase in bonding shear strength. Moreover, in the boundary zone of the sample preheated at room temperature (P-RT), only mechanical bonding was observed, resulting in the lowest bonding shear strength. Conversely, the other samples exhibited a combination of mechanical and metallurgical bonding. Under the preheating temperature of 200 °C, interfacial intermetallic compounds were observed near the center zone, which exhibited the highest bonding shear strength.

## 1. Introduction

Layered metal composites usually possess unique mechanical and chemical properties; thus, they have become a hotspot for research and application [1]. Al/Ti composite plates combine the high thermal conductivity, high plasticity, low specific gravity of Al, and the superior corrosion resistance and high strength at both ambient and high temperatures of Ti. For this, they are widely used in the automobile and aerospace industries, i.e., the skin of aircrafts [2,3,4,5,6,7].

To the best of the authors’ knowledge, the major strategy to fabricate the Al/Ti composite plates thus far insofar is the metallurgy bonding via interdiffusion of Ti and Al at elevated temperature (400–500 °C) and at high pressure. The detailed metallurgy bonding approaches include hot rolling [8,9,10], explosive welding [11,12,13], diffusion welding [14,15,16], and friction stir welding [17,18,19], each of which has its own pros and cons. For instance, explosive welding has the merits of fast production speed and high bonding strength of the Al/Ti interface [12]; however, the explosive process is uncontrollable, and the final product is easily mixed with impurities, which can significantly weaken the properties of the final product. Moreover, diffusion welding is a controllable process, with the product exhibiting a high bonding strength, but it commonly requires high temperature and a long manufacturing time [20]. Therefore, it is necessary to find a new process for the fabrication of Al/Ti composite plates that have the characteristics of high bonding strength, high safety, high controllability, and low cost.

Additive friction stir deposition (AFSD) is an emerging solid-state additive manufacturing process in recent decades and can create fully dense 3-dimentional (3D) components based on deformation metallurgy bonding—rather than melting and solidification—enabling selective-area depositing, repair, and local feature buildup [21,22,23]. During the AFSD process, feedstock is softened by friction heating and extruded into the space between the tool and substrate [24,25]. Then, the extruded deposited material is co-deformed and mixed with the surface layer of the substrate to form a strong interfacial bond [26]. Given that AFSD only takes a few minutes to deposit one single layer, it has characteristics of high controllability, high safety, and high efficiency. Meanwhile, fine-grained microstructure can be obtained in the final product materials due to the severe deformation during AFSD [27]. Consequently, using AFSD, bonding between Al alloy and Al alloy [28,29,30], as well as bonding between Ti alloy and Ti alloy [31,32,33], was performed. However, there is no study reported on the bonding between these two dissimilar metals (Al and Ti alloys) via AFSD.

Additionally, the temperature distribution during AFSD and the mechanical performance of the final product fabricated by a friction stir weld (FSW) has been found to be sensitive to the manufacturing parameters, like the preheating temperature [34]. Since the principle of AFSD is almost similar to FSW, the manufacturing parameters could also influence the temperature distribution during AFSD, as well as the performance of the final AFSD product. For example, in the study of Garcia et al. [35] on Al-Mg-Si and Cu alloys, they reported that the higher the ratio of the square of the rotation speed to the travel velocity, the higher the peak temperature reached during the AFSD process. Phillips et al. [36] reported the positive relationship between the hardness of the material and the manufacturing parameters, including rotation speed, material feed velocity and travel velocity, during the study on Al-Mg-Si alloy using AFSD. Furthermore, Hartley et al. [28] found that the increase in the high rotation speed from 300 revolutions per minute (rpm) to 900 rpm during the AFSD process of Al-Mg-Si alloy produces minor surface defects and additional roughness around the cladding edges. These results reveal that the manufacturing parameters can influence the microstructure and properties of the AFSD product, but a systematic investigation regarding the correlation is still lacking, especially for the unexplored AFSD Al/Ti composite plates.

In this work, the AFSD method was used to bond 6061 Al alloy and TA1 Ti. The impact of preheating temperature on the microstructure and bonding performance was explored. Moreover, a temperature field during AFSD was simulated to study the implication of the preheating temperature for the temperature distribution. According to the study of Stubblefield et al. [25], the temperature can influence the fluidity of the feed material, and also affect the diffusion rate between Al and Ti. Therefore, this study attempts to simulate the temperature field in the experimental process to uncover how temperature influences mechanical properties and interface morphology. Considering the formation of interfacial intermetallic compounds (IMCs) in the samples, the Gibbs energies (Δ*G*) of Ti_3_Al, TiAl_3_, and TiAl were calculated using the thermodynamic technique under various temperatures. The correlation between the preheating temperature of the microstructure and the mechanical properties are also discussed. This study can provide insights on the preparation of Al/Ti composite plates with superior mechanical performance.

## 2. Experimental Section

**Figure 1:**
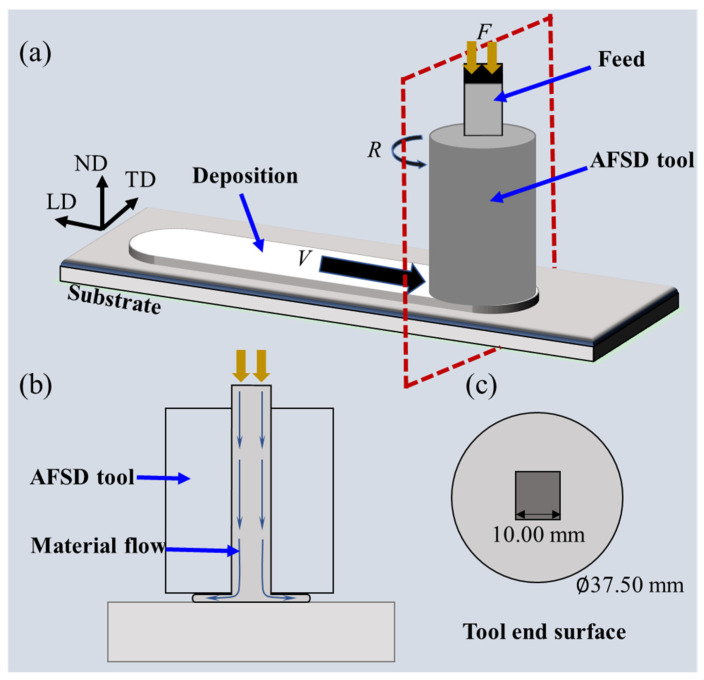
The illustrations of (**a**) the AFSD process, (**b**) the cross-sectional view of the AFSD process, and (**c**) the view of the tool end surface. ND, TD, and LD refer to the normal direction, transverse direction, and longitudinal direction, respectively.

Figure 1 shows the AFSD process of Al/Ti composite plates. As shown in Figure 1a, the key parameters of AFSD includes rotation speed (*R*), material feed velocity (*F*), and travel velocity (*V*). In this work, considering the ultimate tolerance of the equipment, the experimental parameters used in this work were based on the previous experiments [37]. Figure 1c shows the tool surface of the process; note that the flat tool, rather than the protruding tool, was chosen to fabricate the composite plates. This is because the protruding tool will tear and cut the surface of the Ti; thus, the surface of the protruding tool after AFSD is rather rough, which is not suitable for the final product [28]. The first layer of deposition was studied in this work, given its critical influence on the subsequent multilayer deposition.

**Figure 2:**
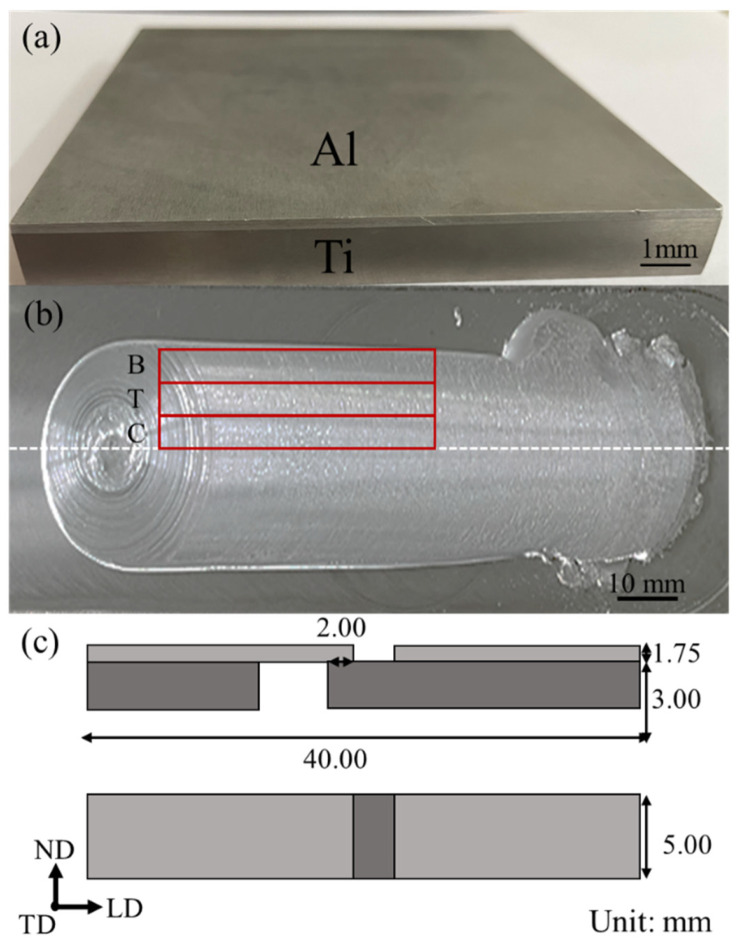
(**a**) Al/Ti composite plates. (**b**) AFSD deposit. It can be divided into three parts: boundary (B), transition (T), and center (C) zones. (**c**) Schematic diagram of the specimens for the shear strength test.

The materials used in this study were a TA1 titanium metal sheet with a thickness of 10 mm and a 6061-T6 aluminum alloy rod with a size of 10 × 10 × 200 mm^3^. The chemical compositions of 6061-T6 and TA1 are tabulated in Table 1. The AFSD was performed on a friction stir additive machine (Central South University, FRC002, Changsha, China) with a rotating speed of 400 rpm, a material feed velocity of 175 mm/min, and a travel velocity of 100 mm/min. The substrate was preheated at room temperature (25 °C, RT), 100 °C, or 200 °C before AFSD, to explore the influence of the preheating temperature on the microstructure and mechanical properties of Al/Ti composite plates. The final 6061/TA1 product after deposition is shown in Figure 2a. The final product was obtained by depositing material in the LD direction, followed by several additional deposits in the TD direction. This study specifically focuses on the product obtained after the first deposition in the LD direction, as shown in Figure 2b. The deposited specimens could be divided into the center (C), transition (T), and boundary (B) zones (Figure 2b). During the deposition, the zone directly affected by the friction of the feed material is defined as the center zone, the boundary of the deposition is defined as the boundary zone, and the region between these two is defined as the transition zone. The nomenclature of the samples herein is the following: P denotes preheating, and it is followed by the preheating temperature (RT, 100 °C and 200 °C), as well as the detailed zone (C, T, B) (e.g., “P-RT-C” stands for “the center zone of AFSD sample prepared under the preheating temperature of RT”). The detailed conditions for samples studied in this work are listed in Table 2. Figure 2c shows the illustration of the shear strength test specimens. The shear strength of each zone was individually measured using a shear strength machine (Chuanbai, Changsha, China) at the strain rate of 0.50 mm/min, based on a standard of GB/T 6396-2008 [38]. To examine the bending performance, three-points bending experiments were performed using the universal testing machine (Instron, Norwood, MA, USA) with the bending specimen dimensions of 2 × 5 × 120 mm^3^, following the standard of YB/T 5349-2014 [39]. The crosshead displacement rate of the bending tests was 3 mm/min. The specimens were pressed by three-points bending on the Al side and the Ti side, individually. The experiment was set to end at a value where the compressive stress dropped 15 percent from the maximum stress. The deflection (*f*) was 20.16 mm and 36.40 mm. Specimens were visually observed with clear cracks or delamination after the bending tests. This supports the observation in tensile tests that the malleability of the substrate material was preserved after the deposition process and that the interface bond was not easily delaminated. Vickers micro-hardness measurements were conducted on LD-ND cross-sections using the HV-5 micro-Vickers (Huaying, Changsha, China). The hardness profiles were generated by performing indentations with total points of 120, where a 5 × 24 grid was divided into a 5 × 30 mm^2^ area for each specimen. Then, each specimen was measured three times, and a standard deviation was calculated for the hardness value at each point. All the standard deviations were in the range of 1 ± 0.5 HV.

The interfacial microstructures of the Al/Ti specimens were characterized using the scanning electron microscope (SEM, FEI 650, Rock Hill, SC, USA) equipped with the energy dispersive spectroscopy (EDS) technique (Rock Hill, SC, USA). The accelerating voltage was 20 kV, the working distance was 16 mm, and the mode for SEM imaging was backscattered electron (BSE). Note that the distance between the point with the min. 20 wt.% Al and the other one with the min. 20 wt.% Ti was selected as the parameter to evaluate the degree of elemental mixture of Al and Ti. An X-ray diffractometer (XRD; Rigku XRD, smartlab SE, Tokyo, Japan) was utilized to identify the phase constitution using Cu Kα radiation with a scanning rate of 2°/min. XRD results were analyzed by Jade 6 software (Materials Data Inc (MDI), version 6.5, Livermore, CA, United States). The morphologies of interface IMCs were also characterized by a transmission electron microscope (TEM; Spectra 300, Thermo Fisher Scientific, Waltham, MA, USA). The round foils for TEM observation were prepared with a standard grinding procedure and then thinning treatment was carried out on the ion milling system (Gatan 695, Pleasanton, CA, USA). The results of TEM and XRD are were presented in the Supplementary Materials Figures S1 and S2.

Additionally, the temperature simulation was performed using Abaqus (Dassault System SIMULIA, Vélizy-Villacoublay, France) to obtain the temperature distributions. The parameters used in this simulation were the same as in our actual experiment, as the rotating speed and the travel velocity were set to be 400 rpm and100 mm/min, respectively. Furthermore, 3 × 10^4^ meshes were generated in the simulation plate, and the elements were set to C3D8RT.

## 3. Results

### 3.1. Microstructure

**Figure 3:**
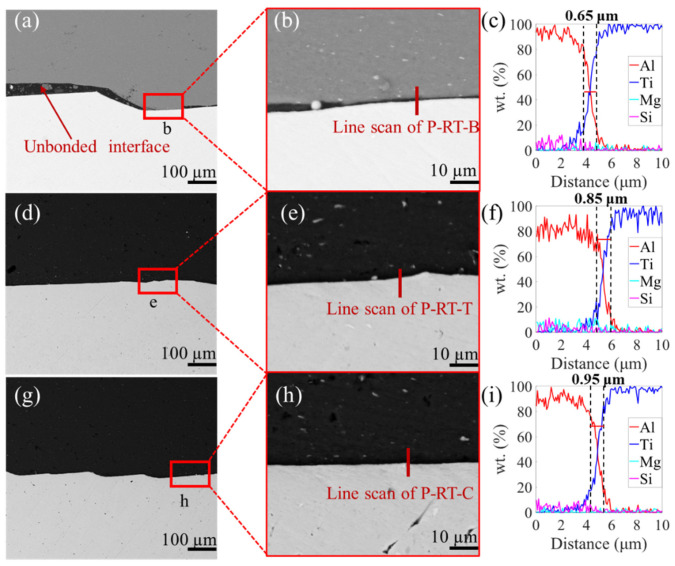
SEM images of P-RT. (**a**) The boundary of P-RT (P-RT-B), and (**b**) the enlarged region b in (**a**). (**d**) The transition of P-RT (P-RT-T), and (**e**) the enlarged region e in (**d**). (**g**) The center of P-RT (P-RT-C), and (**h**) the enlarged region h in (**g**). (**c**,**f**,**i**) are the EDS line scan results of lines in (**b**,**e**,**f**), respectively.

**Figure 4:**
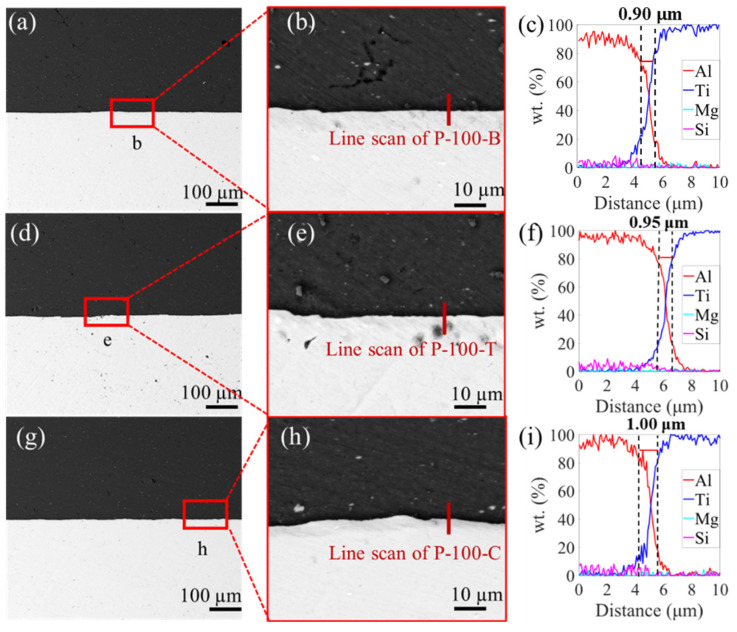
SEM images of P-100. (**a**) The boundary of P-100 (P-100-B), and (**b**) the enlarged region b in (**a**). (**d**) The transition of P-100 (P-100-T), and (**e**) the enlarged region e in (**d**). (**g**) The center of P-100 (P-100-C), and (**h**) the enlarged region h in (**g**). (**c**,**f**,**i**) are the EDS line scan results of lines in (**b**,**e**,**f**), respectively.

**Figure 5:**
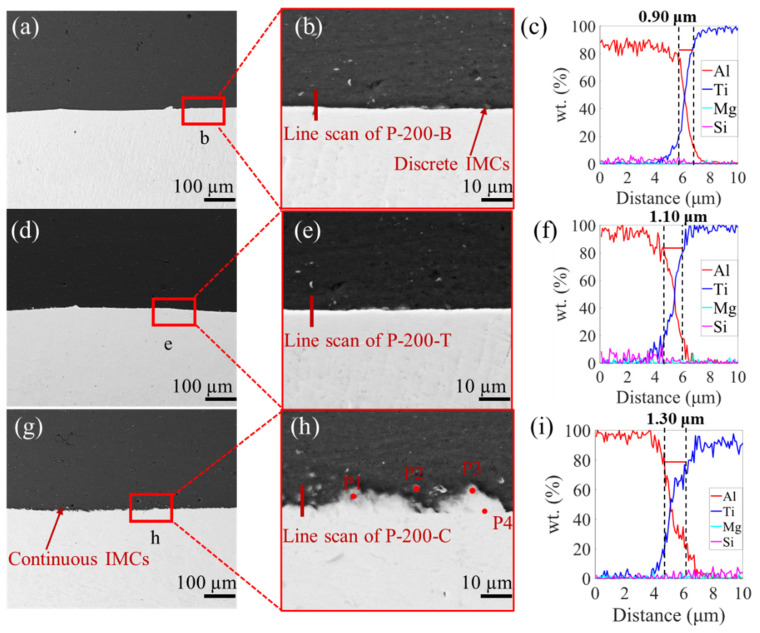
SEM images of P-200. (**a**) The boundary of P-200 (P-200-B), and (**b**) the enlarged region b in (**a**). (**d**) The transition of P-200 (P-200-T), and (**e**) the enlarged region e in (**d**). (**g**) The center of P-200 (P-200-C), and (**h**) the enlarged region h in (**g**). (**c**,**f**,**i**) are the EDS line scan results of lines in (**b**,**e**,**f**), respectively.

**Figure 6:**
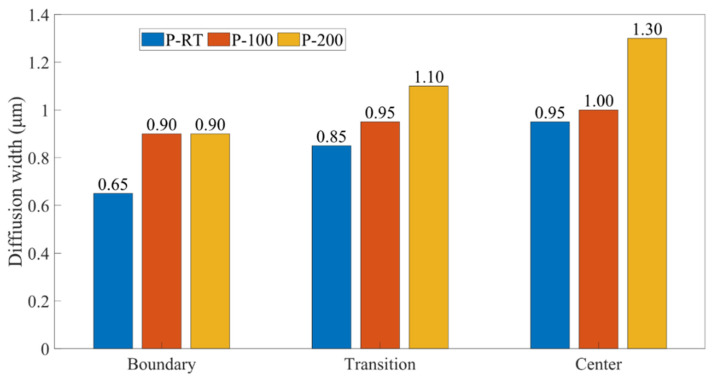
The diffusion width of specimens prepared without preheating (P-RT), as well as with preheating, under the temperatures of 100 °C (P-100) and 200 °C (P-200).

Figure 3, Figure 4 and Figure 5 show the SEM images and the EDS results of the boundary, transition, and center zones under different preheating temperatures. In the P-RT-B, a large unbounded surface, as well as a jagged deformation between the Al and Ti alloys, was clearly observed (Figure 3a,b), indicating this zone suffered severe deformation during AFSD. However, in the P-RT-T and P-RT-C, no visible defects were detected. Moreover, with the progression from the boundary to the center of the AFSD deposit, the elemental mixture distance increased from 0.65 μm to 0.95 μm, suggesting the gradually enhanced interdiffusion of Ti and Al during the AFSD process (Figure 3c,i). A Similar trend was also found in the P-100 and P-200 specimens (Figure 4 and Figure 5). Additionally, unlike P-RT-B, no unbounded surface was observed in P-100-B and P-200-B samples, suggesting a higher preheating temperature is conducive to softening the boundary zone and facilitating the bonding of Al and Ti alloys. Figure 6 quantitatively presents the evolution of the diffusion width in different zones of samples prepared at various preheating temperatures. Two rough basic trends can be observed that: (i) with the transition of the zone from the boundary to the center, the diffusion zone increases; (ii) as the preheating temperature increases, the diffusion zone also increases. Among all the samples, the P-200-C sample has the largest diffusion zone.

It can be also seen from Figure 4 and Figure 5 that the interface formed under preheating temperatures of 100 °C (Figure 4a,b) and 200 °C (Figure 5a,b) was relatively flat, and scarce macro defects were found in the interface. At the Ti-Al interface of P-200-C, many discrete hemispherical regions are found in Figure 5b,g, and they exhibited a different contrast in comparison with the Al and Ti alloys. Given the large deformation and high temperature between Ti and Al during AFSD, these discrete hemispherical regions are postulated to be the Intermetallic Compounds (IMCs) [40]. These IMCs are distributed discretely in the boundary zone and continuously in the center zone. To further explore the elemental constitution of compounds, specimens under a preheating temperature of 200 °C with continuous IMCs were point-scanned at P1, P2, P3, and P4 (cf. Figure 5h); their with whose composition is recorded in Table 3.

**Figure 7:**
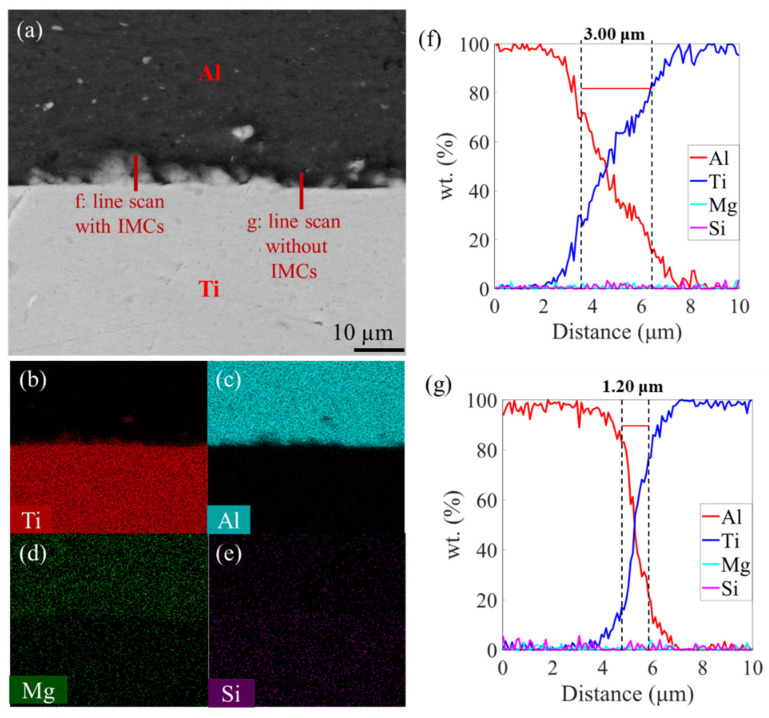
(**a**) SEM picture of the P-200-C specimen. (**b**–**e**) are the EDS map scan results. Line scan results (**f**) with and (**g**) without IMCs from P-200-C.

Meanwhile, the line scan, with and without the IMCs (Figure 7a) and EDS (Figure 7b–e) were performed on the P-200-C. Obviously, these IMCs have the major elements of Al and Ti, in agreement with our hypothesis. Furthermore, an enrichment of the Mg element was found in the Al side, while this element was found to be discretely distributed in the Ti side, based on the EDS results. And, the Si element was found to be discretely distributed in both the Ti and Al sides. This indicates that the diffusions of the Al, Ti, Mg, and Si elements simultaneously occurred during the AFSD process. Figure 7f,g are the results of the line scan of P-200-C, with and without IMCs; the results that without IMCs show the same width as the previous results. The width of the elemental mixture distance reaches 3.00 μm in the specimen whose line scan includes IMCs. Moreover, TEM and XRD was also utilized to analyze the IMCs, and the results are displayed in the Supplementary Materials Figures S1 and S2, respectively. However, unfortunately, they failed to characterize the intermetallic compounds, probably owing to their low fractions and scatter distributions.

### 3.2. Mechanical Behavior

#### 3.2.1. Bonding Strength

**Figure 8:**
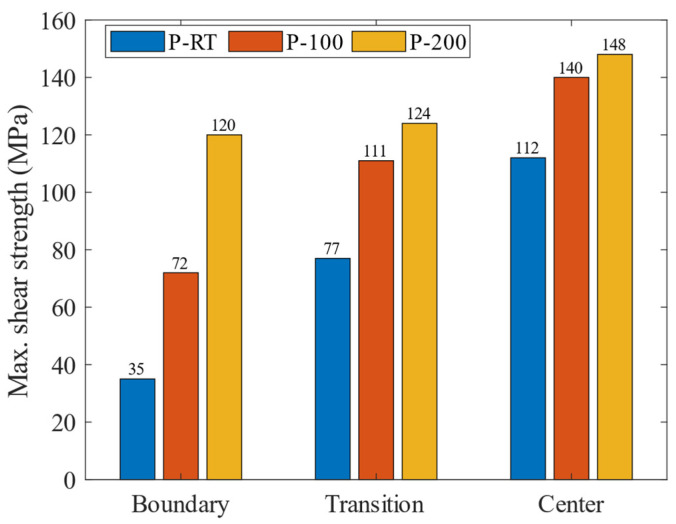
The max. shear strength of specimens prepared without preheating (P-RT), as well as with preheating, under the temperatures of 100 °C (P-100) and 200 °C (P-200).

Figure 8 shows the maximum shear strength of the specimens. It can be clearly seen that the shear strength was gradually increased as the zone evolved from the boundary to the center, and as the preheating temperature increased. The P-RT-B sample has the lowest shear strength of 35 MPa, and the P-200-C sample has the highest shear strength of 148 MPa. Note that in the study of Ma et al. [8], the same Al/Ti composite plate specimens were successfully prepared by the hot rolling technique, and their bonding strength was located around 51–115 MPa, below that of most samples by AFSD in this work.

#### 3.2.2. Three-Points Bending

**Figure 9:**
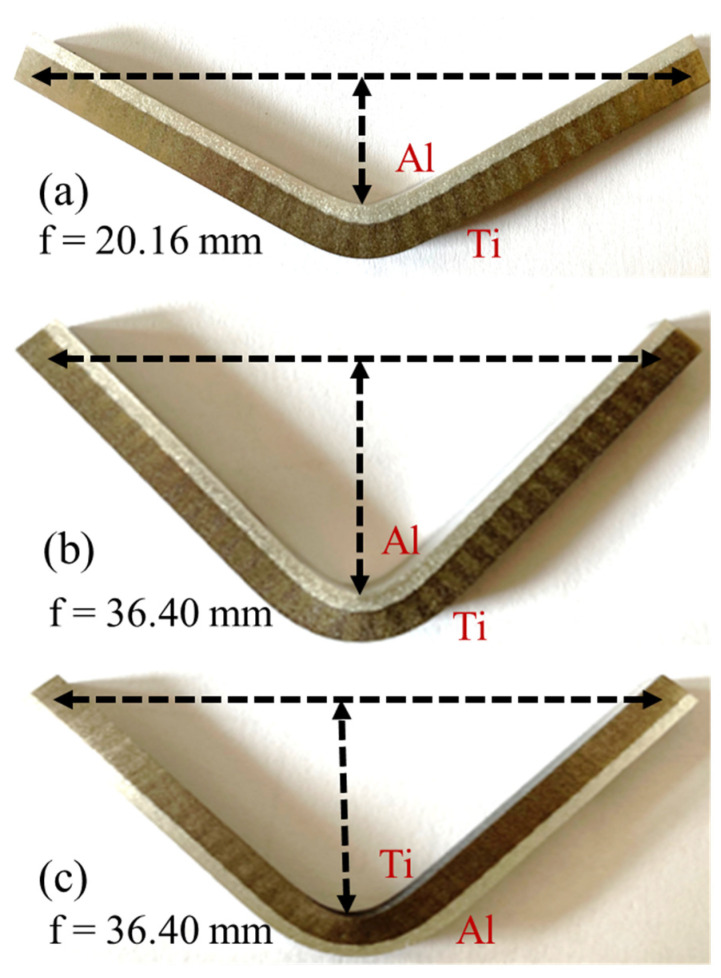
Three-points bending test. (**a**,**b**) Force application roller on Al. (**c**) Force application roller on Ti. The deflection of (**a**) is 20.16 mm; the deflection of (**b**,**c**) is 36.40 mm.

In order to further verify the bonding strength between Ti and Al, a three-points bending experiment was carried out in the present research [28]. The specimens selected were P-RT-B because this sample exhibited the lowest bonding strength (Figure 8). To demonstrate whether cracking or delamination occurs at the interface, the three-points bending test specimen suffered severe deformation. The experimental results of the three-points bending tests are shown in Figure 9. Obviously, under the severe deformation of the three-points bending, no obvious cracking or delamination occurs at the interface, suggesting the superior bonding of the Al/Ti composite plate prepared by the AFSD technique.

#### 3.2.3. Microhardness

**Figure 10:**
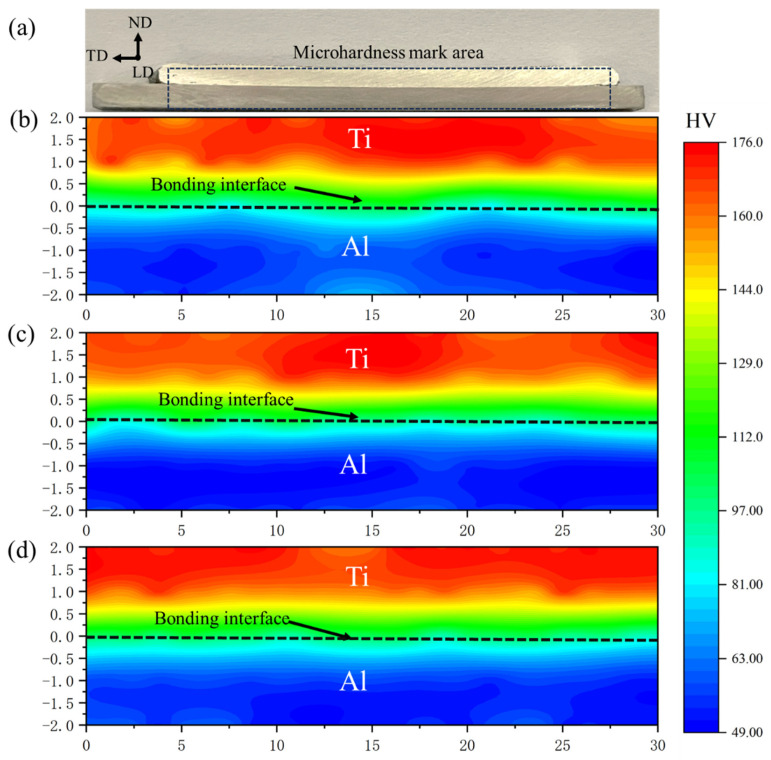
Micro-hardness distribution of Ti/Al. (**a**) The micro-hardness mark area. (**b**) The micro-hardness distribution of P-RT. (**c**) The micro-hardness distribution of P-100. (**d**) The micro-hardness distribution of P-200.

Figure 10 shows the measured area of the Vickers hardness test and the hardness cloud map near the Al/Ti interface. The LD-ND cross-section is shown in Figure 10a. And, the hardness distribution of the P-RT, P-100 and P-200 are presented in Figure 10b–d. The hardness distribution trend is similar at different preheating temperatures. This indicates that different preheating temperatures have no effect on the hardness of the final product. The hardness of the original 6061-T6 alloy was measured to be 115 ± 10 HV, and the hardness of the original TA1 Ti was measured to be 160 ± 15 HV. At the end of deposition, the hardness of the 6061 Al alloy is reduced to 60 ± 15 HV, which is close to that of pure Al material. Like friction stir welding, the decrease of hardness in the deposited material is due to the evolution of Mg-Si precipitates in the process of AFSD. This is ascribed to the dissolution of these precipitates in the Al alloy during AFSD. In the subsequent cooling process, the cooling speed is too high to precipitate it again in the matrix (6061 Al alloy), leading to the decreased Vickers hardness value. In terms of the substrate, while the hardness of TA1 Ti is almost the same as that before AFSD. This is because TA1 Ti is pure Ti, unlike the A6061 Al alloy, which has a strengthening phase, so its hardness value does not change distinctly [41,42].

Moreover, the hardness values gradually decrease from the Ti side to the Al side. The hardness values at the Al/Ti diffusion interface are between that of Ti and Al, ~80–100 HV. This also indicates that the Ti and Al in the AFSD process are not a simple physical combination, but a metallurgical combination formed through diffusion and chemical reaction. According to the hardness studies of friction stir welding [43,44], friction stir welding often leads to hardness differences between the nugget, but the micro-hardness measurements here show that the hardness distribution is still roughly symmetric along the normal direction of the interface. This indicates that the heat generation of AFSD is more symmetrical and uniform. The principle of friction stir welding is almost akin to AFSD. According to the research on friction stir welding, friction stir welding will form a pinhole on the surface, and the production of the composite plate has higher requirements for the welding needle of friction stir welding, and there will be an uneven temperature distribution in the process of friction stir welding.

## 4. Discussion

### 4.1. Temperature Field Simulation

Based on previous results of shear strength, as well as microscopic interfaces, the difference in shear properties and microscopic interfaces at different preheating temperatures and in different deposition zones may be due to different temperature distributions. In the work of Stubblefield et al. [45], they built a model of the temperature field distribution of Al alloys during AFSD. Based on their work, a simplified model was used in this paper to explore the temperature field distribution of Al/Ti AFSD. Finite element analysis (FEA) was used in this work. The temperature simulation was performed using Abaqus standard coupled temp-displacement analysis with the user subroutines DFLUX and DLOAD.

Under the FEA theory of the temperature field, the governing equations and boundary conditions and are as follows [46]:

(1) $$\frac{\partial }{\partial x}\left(k_{x}\frac{\partial T}{\partial x}\right)+\frac{\partial }{\partial y}\left(k_{y}\frac{\partial T}{\partial y}\right)+\frac{\partial }{\partial z}\left(k_{z}\frac{\partial T}{\partial z}\right)+\dot{q}=\rho c\frac{\partial T}{\partial t}\;$$

where *T* is the temperature, and *t* is the time. $k_{x}$, $k_{y}$, $k_{z}$ are heat transfer coefficients, ρ is density; *c* is specific heat; and $\dot{q}$ is the internal heat source intensity.

(2) $$T\left(x,y,z,t\right)=T_{0}$$

(3) $$k_{x}\frac{\partial T}{\partial x}n_{x}+k_{y}\frac{\partial T}{\partial y}n_{y}+k_{z}\frac{\partial T}{\partial z}+q=0$$

(4) $$k_{x}\frac{\partial T}{\partial x}n_{x}+k_{y}\frac{\partial T}{\partial y}n_{y}+k_{z}\frac{\partial T}{\partial z}+h\left(T-T_{\infty }\right)=0$$

where *q* is the boundary heat; *h* is the heat transfer coefficient of the surrounding medium to solid; $n_{x}$, $n_{y}$, $n_{z}$ are the cosines of the outer normal direction on the boundary; $T_{\infty }$ is the surrounding medium temperature. Equation (1) is the governing equations, and Equations (2)–(4) are boundary conditions. In an isotropic material,

(5) $${k_{x}=k}_{y}=k_{z}=k$$

The heat generation source is friction heat generation. During the AFSD process, the power of friction heat generation depends on the friction force and the relative slide between the feed material and the substrate material [47], given by

(6) $$W_{friction}=\int_{slip}\tau \dot{\gamma }dA$$

where $\dot{\gamma }$ is the slip velocity, and τ is the frictional shear stress [48].

(7) $$\tau_{max}=\min\;(\mu P,\sigma_{s}(T)/\sqrt{3})$$

where μ is the friction coefficient; *P* is the contact pressure; $\sigma_{s}$ is the yield strength, which is related to the current temperature. It can be seen from the flow stress curve of A6061 aluminum alloy (not given in this manuscript) that τ can be calculated by the penalty function, up, when the temperature is less than 300 °C, but when the temperature is higher than 300 °C, τ is expressed by the shear force [49]. Equations (6) and (7) were translated into Fortran code and compiled to the DFLUX and DLOAD subroutine.

The process of AFSD was divided into two steps. Step-1 was set so that the tool was rotated by the AFSD machine, but the tool was not moved. Due to the friction of the feed material and substrate, the temperature of the contact interface between the feed material and the substrate gradually increased, which caused the feed material to enter the rheological state [50]. After the feed material in Step-1 started to deposit on the substrate material, Step-2 began. The tool maintained the same rotation speed as Step-1 and started to move. Feed material was deposited on the substrate along the tool moving path. In the actual experiments the AFSD process Step-1 at room temperature takes more time than preheating conditions. So, in the simulation for room temperature, the time of Step-1 is longer than others.

**Figure 11:**
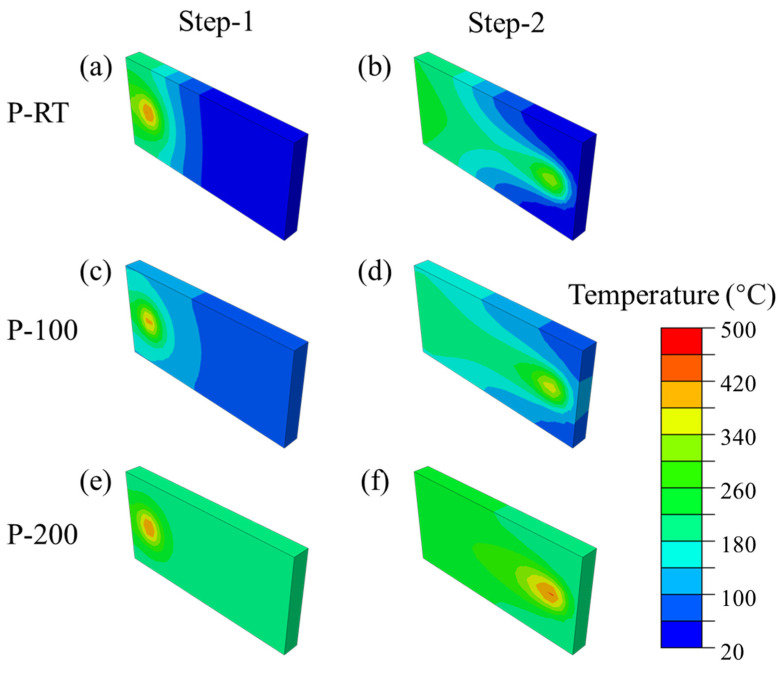
AFSD temperature field simulation. Step-1 is that the rod rotates, and friction heat generates. Step-2 is the end of deposition. (**a**) RT-Step-1 (**b**) RT-Step-2 (**c**) 100 °C-Step-1, (**d**) 100 °C-Step-2, (**e**) 200 °C-Step-1, (**f**) 200 °C-Step-2.

The temperature field simulation results are displayed in Figure 11. Figure 11a,c,e show the temperature distribution of the RTs, 100 °C and 200 °C, in Step-1; with the increase in the preheating temperature, the temperature distribution of Step-1 becomes more uniform, and the average temperature also increases with the increase in the preheating temperature. Therefore, in Step-2, Al can be better softened, which causes better flowability. Figure 11b,d,f show the simulation results of Step-2. From Figure 11b,d, the peak temperature in Step-2 is lower than in the corresponding Step-1. The reason why the peak temperature at the end of Step-2 is less is because that heat transfer and radiation occur between the feed material, the substrate material, and the external medium (such as the air and the tool) in the process of Step-2. Since the friction force does not follow the penalty function rule in the high-temperature environment, the τ in Equation (6) decreases, and the heat production decreases accordingly. Meanwhile, the moving area of Step-2 is larger, and so is the heat conduction area of the substrate. These are all possible reasons for the lower peak temperature in Step-2. Given that the center zone has the highest temperature, the rheological behavior of the feed material is more likely to occur in the center zone. Moreover, the higher the temperature, the faster the diffusion between the Al alloy and Ti [51], and the easier it is to form a better metallurgical bonding area.

### 4.2. Correlation among Preheating Temperature, Microstructure, and Mechanical Strength

According to the line scan results in Figure 3, Figure 4 and Figure 5, the width of the elemental mixture distance gradually widens from the boundary to center zones under the same preheating temperature. And, the width of the elemental mixture distance also increases with the enhancement of the preheating temperature in the same zone. Combined with the temperature field simulation of different preheating temperatures in Figure 11, it can be seen that the higher the temperature in the AFSD process, the wider the elemental mixture distance obtained. The difference in temperature leads to different diffusion rates, as well as the different width of the elemental mixture distance.

In Figure 3, Figure 4 and Figure 5, compared to the interfaces of the P-100 and P-200, the interface of P-RT has obvious mechanical deformation, which causes the interface to be very uneven. The reason for the uneven interface is that Ti and Al are still hard due to the insufficient temperature, and the contact face of Al will tear and cut the surface of Ti under the high pressure from the AFSD machine. Combining the previous analyses, a large deformation and unbound area were found in P-RT-B; thus, it can be speculated that the combination mode of P-RT is mechanical bonding [52]. For the P-RT-C and P-RT-T, the interface was also uneven, but no unbound area was observed. Combining the line scan results of the P-RT-C and P-RT-T, it can be found that the combination mode of these two should be the combination of mechanical and metallurgy bonding. In P-RT, the different combination mode and different element distance lead to the difference in the shear strength [14]. In the P-100, the interface is flat, and the element mixture distance increases from the boundary to the center, which causes the difference of the shear strength in P-100.

Given that IMCs only appear in P-200, it is also necessary to discuss its formation during the AFSD process, as well as the influence of the preheating temperature on it. According to the study of Thiyaneshwaran et al. [20] and the binary phase diagram of Ti-Al (not given in this manuscript), the mesophase compounds formed by Ti-Al are TiAl, Ti_3_Al and TiAl_3_ at a melting temperature lower than that of aluminum, and their Gibbs free energy expressions are shown as follows:

(8) $$∆G_{{Ti}_{3}Al}=-29,663.6+6.70801T$$

(9) $$∆G_{{TiAl}_{3}}=-40,349.6+10.3625T\;$$

(10) $$∆G_{TiAl}=-37,445.1+16.79376T$$

**Figure 12:**
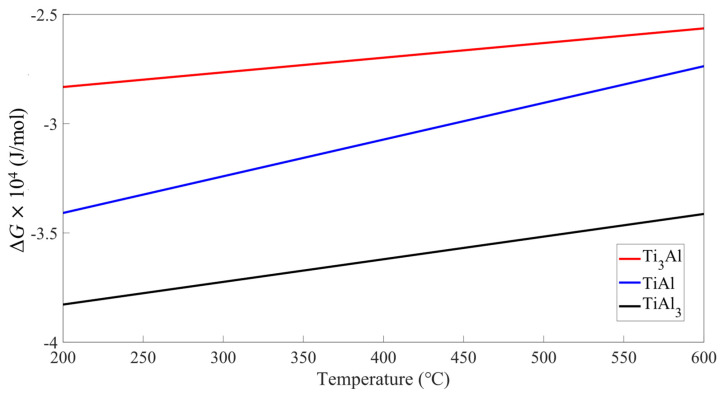
Gibbs free energy (Δ*G*) of Ti_3_Al, TiAl_3_, and TiAl with respect to the temperature.

Gibbs free energy curves of the three compounds were plotted and are shown in Figure 12. From it, the Gibbs free energy of TiAl_3_ is the largest in the experimental temperature range of AFSD. It can be considered that TiAl_3_ is easier to form. From the study of Song et al. [40], high-temperature agitation between Ti and Al makes it easy to have particles on the titanium surface peel off and mix into the interface between Ti and Al, and it is easy to have intermetallic compounds between Ti and Al being generated. According to the study of Wei et al. [14], the strengthening principle is as follows: Ti-Al compound is formed during the process of diffusion between the Ti and Al interfaces. Ti -Al compound and Ti particles are dispersed and cannot be connected to each other, so dislocation of Al and Ti will occur at the interface. This results in greater shear strength in the area with these IMCs. Li et al. [53] reported that continuous lamellar or serrated IMCs will improve the tensile strength of the Al/Ti. Zhao et al. [54] reported that in Al/Ti composite plates prepared by friction stir welding, the IMCs and diffusion layer at the interface will enhance the shear strength and elongation of the interface. These are in good agreement with this work, in that the P-200-C sample with IMCs near the Al/Ti interface exhibits the highest shear strength among all the samples.

## 5. Conclusions

Additive friction stir deposition (AFSD) was successfully applied to prepare Al/Ti composite plates with good surface quality, but without local buckling or wrinkling. The influence of preheating temperatures (room temperature, 100 °C, 200 °C) on the interfacial microstructure and interface mechanical properties at different zones of deposition was also investigated. The main conclusions can be drawn as:

1. Both the diffusion width of the Al/Ti interface and the resultant bonding shear strength increase with the increase in the preheating temperature, and also as the zone progresses from the boundary to the center of the deposit. And, the center sample prepared at the preheating temperature of 200 °C exhibits the largest diffusion width as well as the highest bonding strength. A positive correlation between the diffusion width of the interface and the bonding shear strength is found.
2. In terms of the bonding mechanism, in P-RT, it is the mechanical bonding in the boundary zone that dominates, while in the other samples, the combined mechanical and metallurgy bonding dominates.
3. The simulated temperature field distribution of AFSD between Al and Ti reveals that during the deposition process, the temperature constantly changes from the center to the edge. This accounts for the different widths of the elemental mixture distance and the difference of the shear strength in different zones.
4. The appearance of interfacial intermetallic compounds (IMCs) could be ascribed to the high temperature near the center zone, high preheating temperature, and low Gibbs free energy of TiAl_3_. Due to the dispersed characteristic of IMCs, their presence increases the shear strength of the Al/Ti composite plate.

## Figures and Tables

**Table 1:** Chemical compositions of commercial A6061 and TA1 (wt.%).

	Al	Mg	Si	Ti	Fe	Mn	Cu	Zn
6061	Bal.	1	0.8	0.1	0.7	0.1	0.2	0.2
TA1	-	-	-	Bal.	0.2	-	-	-

**Table 2:** Summary of nomenclature and corresponding conditions for various samples.

	Preheating at Room Temperature	Preheating at 100 °C	Preheating at 200 °C
Center	P-RT-C	P-100-C	P-200-C
Transition	P-RT-T	P-100-T	P-200-T
Boundary	P-RT-B	P-100-B	P-200-B

**Table 3:** Elemental analysis of P1 P2 P3 and P4 of Figure 5h.

	P1	P2	P3	P4
Ti (wt.%)	28.8	15.9	36.2	32.4
Al (wt.%)	70.3	83.4	62.9	66.0
Mg (wt.%)	0.5	0.5	0.5	0.9
Si (wt.%)	0.4	0.2	0.4	0.7

## Data Availability

Data available on request from the authors.

## Supplementary Materials

The following supporting information can be downloaded at: https://www.mdpi.com/article/10.3390/ma16176018/s1, Figure S1: TEM result of P-200-C sample. (a) bright field TEM image, and (b) enlarged region in (a). (c) Al, (d) Mg, (e) Si and (f) Ti EDS map scan results of (b); Figure S2: XRD result of the P-200 sample in various zones. Only peaks corresponding to fcc-Al and hcp-Ti were identified.
