# Supplementary material for: Influence of Preheating Temperature on the Microstructure and Mechanical Properties of 6061/TA1 Composite Plates Fabricated by AFSD
Source: Materials (Basel). 2023 Sep 1;16(17):6018. doi: 10.3390/ma16176018 (PMC10488640; doi:10.3390/ma16176018)
Supplement: Supplementary file 1 [file materials-16-06018-s001.zip › materials-2548236-supplementary.pdf]

Supplementary materials of “Influence of preheating temperature on the microstructure and mechanical properties of 6061/TA1 composite plates fabricated by AFSD”

Wei Gong, Yidi Li, Ming Zhang, Hui Wang, Qinglin Liu, Ziming Zeng, Kuo Ma, Biaobiao Yang, Ruilin Lai, Yunping Li

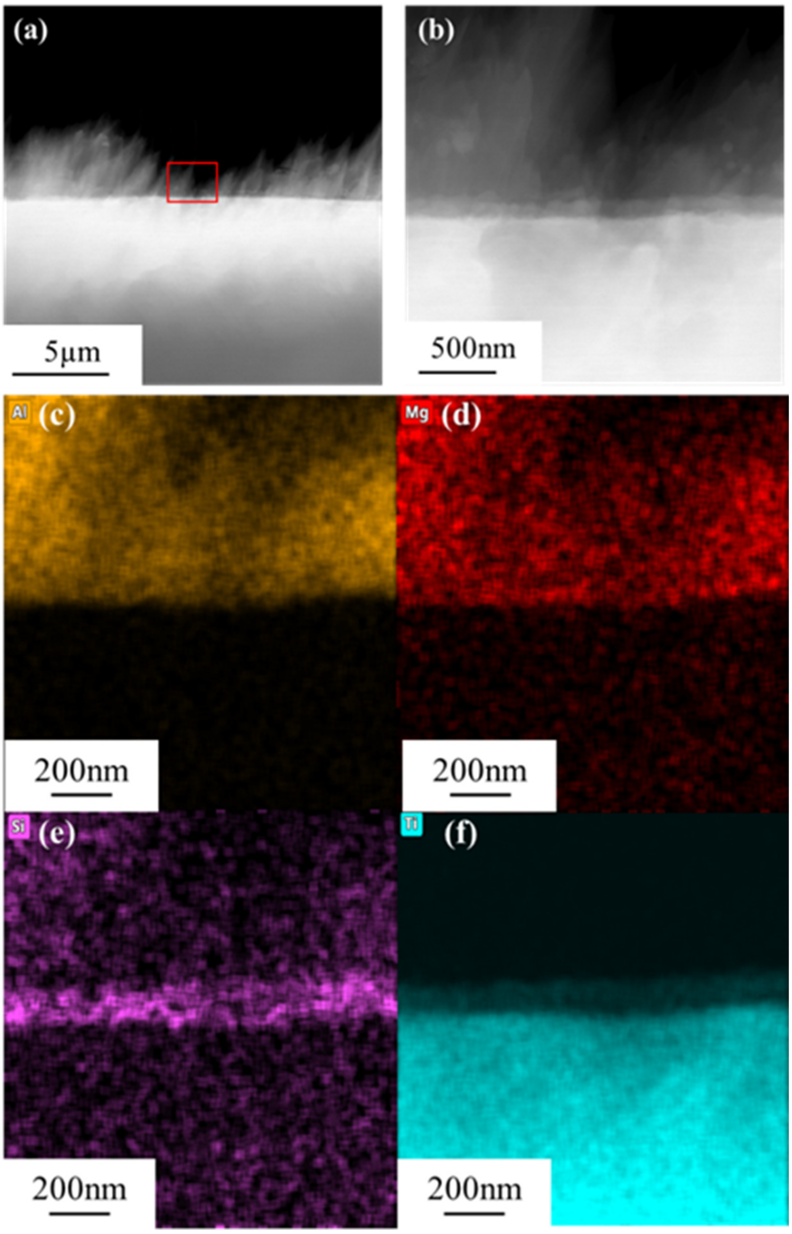

Fig. S1. TEM result of P-200-C sample. (a) bright field TEM image, and (b) enlarged region in (a). (c) Al, (d) Mg, (e) Si and (f) Ti EDS map scan results of (b).

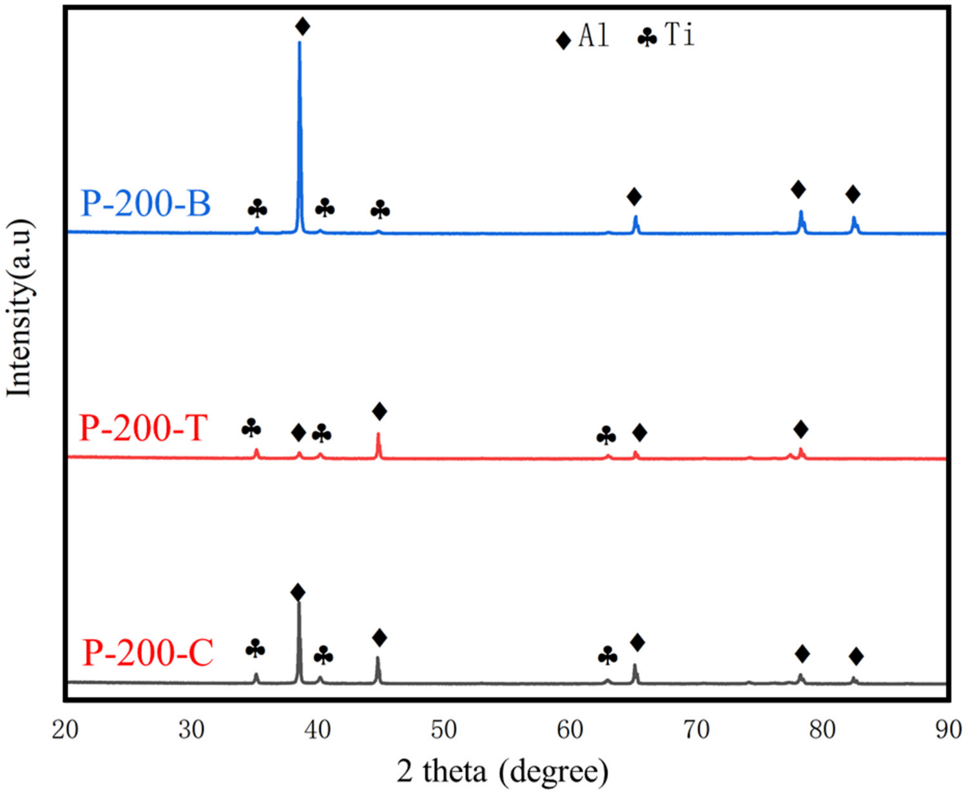

Fig. S2. XRD result of the P-200 sample in various zones. Only peaks corresponding to fcc-Al and hcp-Ti were identified.
